# Five-week yin yoga-based interventions decreased plasma adrenomedullin and increased psychological health in stressed adults: A randomized controlled trial

**DOI:** 10.1371/journal.pone.0200518

**Published:** 2018-07-18

**Authors:** Daiva Daukantaitė, Una Tellhed, Rachel E. Maddux, Thomas Svensson, Olle Melander

**Affiliations:** 1 Department of Psychology, Lund University, Lund, Sweden; 2 Department of Clinical Sciences, Lund University, Lund, Sweden; 3 Clinical Biotechnology, Center for Disease Biology and Integrative Medicine, Graduate School of Medicine, University of Tokyo, Tokyo, Japan; 4 Department of Internal Medicine, Skåne University Hospital, Malmö, Sweden; TNO, NETHERLANDS

## Abstract

**Background:**

Non-communicable diseases (NCDs, e.g. cardiovascular disease) are responsible for high rates of morbidity and the majority of premature deaths worldwide. It is necessary to develop preventative interventions that can reduce the associated risk factors of NCDs. Researchers have found that the biomarker adrenomedullin (ADM) becomes elevated years before the onset of NCDs and might play an important role in their development. ADM has also been linked to psychological problems such as stress, anxiety, and depression, which are known risk factors of NCDs. In this randomized controlled trial, we examined whether participating in a five-week yoga intervention reduces ADM and increases psychological health in middle-aged adults who self-report as moderately to highly stressed, but who otherwise exhibit no physical complaints.

**Methods:**

One hundred and five adults (78% women; mean age = 53.5, SD = 6.7) were randomly assigned to (1) a five-week Yin yoga intervention, (2) a five-week intervention combining Yin yoga with psychoeducation and mindfulness practice (called the YOMI program), or (3) a control group who did not practice yoga or mindfulness for five weeks.

**Results:**

Compared to the control group, we observed significantly greater pre-post reductions in plasma ADM levels (*p* < .001), anxiety (*p* ≤ .002), and sleep problems (*p* ≤ .003) in both intervention groups. Furthermore, the YOMI group exclusively showed significantly greater pre-post reductions in stress (*p* = .012) and depression (*p* = .021) compared to the control group. Significant correlations (*p* < .05) were found between pre-post reductions in ADM and anxiety symptoms (*p* = .02) and depression (*p* = .04) in the entire sample.

**Conclusion:**

The five-week Yin yoga-based interventions appeared to reduce both the physiological and psychological risk factors known to be associated with NCDs. The study suggests that incorporating Yin yoga could be an easy and low-cost method of limiting the negative health effects associated with high stress.

**Trial registration:**

ClinicalTrials.gov NCT03428542

## Introduction

Non-communicable diseases (NCDs), especially cardiovascular disease (CVD) and cancer, are responsible for high morbidity rates and the majority of deaths worldwide [1]. Besides well-known preventative interventions recommended in the healthcare system, such as smoking cessation, improved dietary habits, and increased physical activity (e.g., [2, 3]), practicing y*oga* has been the subject of recent interest as a possible way of improving the risk factors associated with NCDs; accordingly, medical professionals are debating whether yoga practice should be included in future health recommendations [4]. In this study, we examined the effects of two Yin yoga-based interventions on the vasoactive peptide adrenomedullin (ADM) and common psychological risk factors of NCDs, including stress, anxiety, depression, and insomnia [5, 6].

ADM is secreted from many organs, including the endothelium. It mediates vasodilation, angiogenesis, and growth modulation, and thus exerts both growth-promoting and growth-inhibitory effects on cells [7–9]. Importantly, plasma ADM levels are elevated years before the onset of several major NCDs [10, 11]–particularly, high concentrations in healthy individuals strongly and independently predict the later development of CVD and cancer, as well as premature mortality [10, 11]. In acute conditions of vascular and cardiac stress, ADM is one of the strongest predictors of death known to date [12, 13]. Although there are no agreed-upon thresholds for plasma ADM concentrations, measurements in healthy control subjects indicate that normal concentrations are 1–10 pM [9]. Increased ADM concentrations may be a compensatory mechanism of elevated blood pressure [9]. ADM concentrations also increase with body mass index (BMI) [14, 15] and are elevated in people with chronic conditions, such as congestive heart failure, hypertension, and renal disease [5]. However, ADM concentrations might rise without concomitant pathology, such as during exercise [5], or rapidly change during orthostatic challenges [6].

While there is much evidence linking ADM with stress, anxiety, and depression, it is still premature to conclude that these relationships are causal. In a study on rodents, stress stimuli known to increase sympathetic activity led to elevated ADM in the pituitary gland, plasma, and adrenals, suggesting that it modulates hypothalamic-pituitary-adrenal axis activity [16]. Elevated ADM was found in patients with major depression [17], and according to a large population-based study, plasma ADM partly explains the relationship between depressive symptoms and mortality risk during long-term follow-ups [18]. The results have been mixed for anxiety disorders, with one study finding elevated ADM levels in such patients [19], but another finding reduced levels [20]. Thus, more research is needed.

Yoga is an umbrella term for various physical, mental, and spiritual practices originating in ancient India. In Western society, its best-known form is Hatha yoga, which generally entails performing sequences of physical postures combined with deep breathing in a controlled and mindful manner [21]. Mindfulness, which Kabat-Zinn [22] refers to as “paying attention in a particular way: on purpose, in the present moment, and non-judgmentally” (p. 4), is an essential aspect of yoga practice. Yin yoga, the basis for the interventions examined in this study, is a calmer, more meditative form of yoga that uses seated and lying postures, held for three to five minutes, while maintaining deep breathing [23, 24]. It is derived from Hatha yoga. Its focus on calmness and mindfulness makes Yin yoga a tool for relaxation and stress coping, thereby improving psychological health [24, 25]. As such, Yin yoga may help lower perceived levels of stress and anxiety, which in turn may reduce the physiological and psychological risk factors associated with NCD.

Yoga can improve various psychological-health-related factors, including stress, anxiety, depression, and sleep problems [26–28], all of which are associated with NCDs [29–32]. While practicing yoga may not reduce the challenges, or *stressors*, of daily life, it may provide tools for coping with them, thereby reducing the negative health impacts of stress [33]. The YOMI program, which combines Yin yoga with psychoeducation, produced significant pre-post reductions in stress and worry among young moderately to highly stressed adults [25]. In line with this previous study [25], we expected this combination of Yin yoga with psychoeducation to have stronger health-beneficial effects than Yin yoga alone.

This randomized controlled trial tested the following hypotheses: (1) five-week interventions combining Yin yoga with psychoeducation (the YOMI program), and Yin yoga practice alone, reduce levels of ADM, perceived stress, anxiety, depression, and sleep problems to a greater extent than a control group, and (2) pre-post changes in ADM are related to pre-post changes in the psychological health variables.

## Method

### Design

This study was conducted as a three-group, parallel randomized controlled trial. Randomization was conducted at the level of individual participants by research staff who were blinded to study interventions and hypotheses, using the web-based tool Research Randomizer [34]. The CONSORT checklist (S1 File), the Project protocol (S2 File) and the Data file (S3 File) are provided as supporting information.

### Participants

Participants were recruited from the general population in southern Sweden November 20—December 15, 2015, through advertisements in local newspapers. These advertisements called for people experiencing chronic stress in everyday life. People who registered for the study were screened via telephone interviews for inclusion by research staff not involved in either the data collection or yoga interventions. The inclusion criteria were as follows: (1) experiencing moderate to high stress in everyday life for the past month (i.e. had a total score of 8 or higher [on a range of 0 to 16] on the 4 selected items from the Perceived Stress Scale [PSS]) [35], (2) being physically fit enough (based on a self-report) to perform slow but deep yoga postures, (3) could participate during the intervention period, and (4) aged 40–65 years. The exclusion criteria were (1) previous regular yoga or mindfulness practice (i.e. more than 6 months of practice in the past year), (2) current psychological or psychopharmacological treatment, or (3) inability to attend more than 5 of the scheduled 10 yoga sessions.

Of the participants assessed for eligibility (*n* = 200), 105 met the inclusion criteria and were subsequently randomly assigned to one of the three conditions described below. Table 1 presents the descriptive statistics for the full baseline sample and for each intervention group.

**Table 1:** Descriptive statistics of the participants at baseline.

Variable	Group			p values
	YOMI (n = 33)	Yin Yoga (n = 34)	Control (n = 30)	
Sex (women, n %)	26 (79%)	27 (79%)	23 (77%)	0.96
Age (M±SD)	54.4 ± 7.0	53.4 ± 5.7	52.6 ± 6.8	0.56
Education (n %)				0.64
High school degree	11 (33.3%)	15 (44.1%)	12 (40.0%)	
Bachelor’s/Master’s degree	19 (57.6%)	19 (55.9%)	17 (56.7%)	
Other	2 (6.1%)	0 (0%)	1 (3.3%)	
Marital status (n %)				0.47
Single	9 (27.3%)	10 (29.4%)	5 (16.7%)	
Married/co-habiting	24 (72.7%)	23 (67.7%)	25 (83.3%)	
Other	0 (0%)	1 (2.9%)	0 (0%)	
Employment status (n %)				0.73
Full/part-time job	28 (84.8%)	29 (85.3%)	27 (90%)	
Unemployed	1 (3%)	2 (5.9%)	2 (6.7%)	
Other	4 (12.1%)	3 (8.8%)	1 (3.3%)	
Body mass index (M (kg/m^2^) ± SD)	25.6 ± 4.2	25.3 ± 5.0	26.1 ± 4.2	0.78
Cystatin C (mg/L) (M±SD)	0.88 ± 0.1	0.84 ± 0.1	0.90 ± 0.2	0.10
ADM^#^ (M±SD)	6.1^a^^,^^b^ ± 0.3	5.8^a^ ± 0.3	5.9^b^ ± 0.4	0.01
Perceived stress (M±SD)	20.6±5.9	19.3±5.7	19.2±6.2	0.55
Anxiety (M±SD)	11.3^a^±3.6	10.3±3.6	8.9^a^±4.1	0.05
Depression (M±SD)	6.4±3.3	6.2±2.9	5.6±3.3	0.61
Insomnia (M±SD)	20.2±5.9	20.7±6.2	18.1±6.7	0.23

*Note*. ADM = adrenomedullin.

^#^Expressed as a relative concentration, NPX units.

^a,b^Means sharing the same superscript are significantly different between the groups.

### Interventions

The participants (n = 105) who met the inclusion criteria were randomly assigned to one of three conditions:

1. Yin yoga intervention: Engaged in 60 minutes of Yin yoga practice, twice a week for five consecutive weeks
2. YOMI program [25]: Engaged in 60 minutes of Yin yoga practice and 30 minutes of psychoeducation and mindfulness practice, twice a week for five consecutive weeks. For details on the YOMI program sessions, see S1 Table.
3. Control group: No yoga or psychoeducation for five weeks; then, following post-test measurements, they participated in a three-hour Yin yoga, psychoeducation, and mindfulness practice workshop.

Yin yoga is gentle and practicable for most people, regardless of physical fitness level [23, 24]. It is available in most urban areas of the developed world.

The YOMI program is organized into 10 sessions, each with its own theme. The theme is introduced first, followed by 30 minutes of psychoeducation combined with mindfulness practice. Subsequently, participants practice 60 minutes of Yin yoga, allowing the psychological theme to be further explored, aided by verbal instructions of the yoga teacher (for details, see [25]; see also S1 Table for a content summary of each session).

The interventions were led by two licensed clinical psychologists who were also trained yoga instructors. They were hired as independent yoga instructors for this study and blinded to the study details, including the hypotheses and measures. The interventions were delivered between February 1 and March 18 2016, and took place in a local meeting room facility at no cost to participants. The pre-intervention (baseline) assessment was performed about a week (Yin Yoga group: January 18–29, 2016, YOMI group: February 1–12, 2016) before the first intervention session, while the post-intervention assessment was conducted within approximately a week after the interventions were finished (Yin Yoga group: Mars 7–18, 2016, YOMI group: Mars 21 –April 1, 2016).

In addition to the interventions, the yoga groups were given daily breathing assignments. They were provided a CD containing a 10-minute voice recording called *Conscious Breathing*, comprising guided instructions that encouraged participants to focus on their breath and to use calm, slow nostril breathing. The purpose of the CD was to help participants learn and incorporate deliberate breathing, one of the main elements of yoga and mindfulness, into their everyday life. Participants in both intervention groups were asked to record the time in minutes they practiced the daily breathing assignment, and to hand in the sheets on which they recorded this information after the interventions.

Participants randomized to the control group completed the pre- and post-intervention assessments about 1 week before (February 15–26, 2016) and after (April 4–16, 2016), respectively, the waiting period. They were instructed to not practice any yoga or mindfulness during the five-week period. As an incentive, they were offered a free, three-hour yoga workshop led by the instructors after the post-intervention assessment. All participants in the study received a free yoga mat as compensation for their participation.

### Ethical considerations

The study was approved (2015-10-07) by the Regional Ethical Review board at Lund University, Sweden, before patient recruitment began. All participants were informed of the study’s aims and their right to discontinue participation at any time without penalty. Written informed consent was obtained from all participants. To ensure participant confidentiality, all identifying information was recorded on a single, securely located master list. Each participant was assigned a numeric code used for data analysis. Measures were administered by trained medical staff at the local hospital, who were blinded to the study’s hypotheses.

The trial was retrospectively registered with ClinicalTrials.gov under the title, “The Effects of Yoga on Psychological and Physiological Health in a Population Based Cohort” (number NCT03428542). The registration was made retrospectively, because we were uncertain as to whether we needed to register on ClinicalTrials.gov, since it is an American organization and this study was conducted in Sweden. We confirm that all ongoing and related trials for this intervention are registered.

### Measures

Demographic information as well as anthropometric data and blood tests (including cystatin C) were collected at the start of the intervention. Physiological measures of ADM and self-reported psychological measures were collected at both the pre- and post-intervention assessments.

#### Physiological measure

**Adrenomedullin.** Fasting serum and plasma blood samples were collected via venipuncture; a total of 65 ml of blood (3 tubes) was collected for each participant. Blood samples were immediately frozen to -80°C. Plasma concentrations of ADM were quantified using a validated high-specificity immunoassay, the Proximity Extension Assay, which has been described in detail elsewhere [36, 37].

#### Psychological measures

**The Perceived Stress Scale (PSS).** The PSS [35] is a 10-item scale (e.g., “In the last month, how often have you felt that you were unable to control the important things in your life?”) measuring the degree to which one appraises situations in one’s life as stressful. Items are scored from 0 (never) to 4 (very often), and the total score was calculated as the sum of all 10 items. This scale has shown good internal consistency, with Cronbach’s alpha coefficients ranging from .80 to .86 [38]. In this study, the Cronbach’s alpha coefficient was .85 at pre-intervention (T1) and .80 at post-intervention (T2).

**The Hospital Anxiety and Depression Scale (HADS).** The HADS [39] is a 14-item scale used to assess levels of anxiety (7 items; e.g. “I get a sort of frightened feeling as if something awful is about to happen”) and depression (7 items; e.g. “I feel as if I am slowed down”). Items are scored from 0 (not at all) to 3 (most of the time), with the item scores of each subscale being summed to obtain total subscale scores. The psychometric properties of the HADS are well-established, with average Cronbach’s alpha coefficients for the anxiety and depression subscales of .83 and .82, respectively [40]. In the present study, the Cronbach’s alpha coefficients were .78 (T1) and .84 (T2) for anxiety, and .79 (T1) and .84 (T2) for depression.

**The Insomnia Severity Index (ISI).** The ISI [41] was designed as both a brief screening measure for insomnia and an outcome measure for use in intervention research. The index contains 7 items (e.g., “How worried/distressed are you about your current sleep problem?”) including severity of sleep onset and maintenance (e.g. middle and early morning awakening) difficulties, satisfaction with current sleep pattern, interference with daily functioning, appearance of impairments attributed to sleep problems, and the degree of concern caused by insomnia. The items are rated on a five-point Likert scale from 0 (not at all) to 4 (extremely), and the total score is calculated by summing all 7 items. It has shown good internal consistency (Cronbach’s alpha of .76 [42]). The Cronbach’s alpha coefficients in this study were .90 at both T1 and T2.

### Statistical analyses

Between-group comparisons of the baseline characteristics were conducted using analysis of variance (ANOVA) and independent-sample t-tests for continuous variables and chi-square tests for categorical variables. Pearson correlations were calculated among the outcome variables at baseline, as well as between pre-post changes in ADM and pre-post changes in the psychological outcome measures. All the above-mentioned analyses were performed with SPSS Statistics 24. Preliminary data checks were conducted to ensure there were no violations of the assumptions of linearity, normality, and homogeneity of variances.

The effects of the intervention on the main study outcomes were investigated by estimating models with the full-information maximum likelihood (FIML) method in SPSS AMOS, which allows analyses of all available data (i.e. without excluding individuals who did not complete the study). The FIML approach outlined by Hartley and Hocking [43] does not impute missing data; instead, it obtains parameter estimates by maximizing the likelihood function of the incomplete data using all the information already contained in the incomplete dataset. Outcome variables were all study variables calculated as the difference between pre-intervention and post-intervention scores, and the predictor was the dummy-coded group variable (1 = experimental, 0 = control) and relevant controls, including age (continuous) and participant sex (1 = female, 0 = male). Analyses with ADM as the dependent variable were further adjusted for BMI and pre-intervention kidney function (i.e. levels of cystatin C at T1) because of the known association between elevated plasma ADM concentrations and high BMI [14, 15] and renal disease [5].

G*Power, 3.1.7 [44] was used to calculate *a priori* power for analysing the main effects. Assuming a small effect size (f = 0.15) [45], a significance level (α) of .05, and the power values of the *F* tests of .95, a total of 89 participants had to be recruited. Referring to a previous study [25], we estimated attrition as approximately 15%. Thus, to attain the required sample size for these power estimates, including expected attrition, a total of 105 participants had to be recruited.

## Results

### Descriptive statistics

The baseline characteristics are shown in Table 1. There were no statistically significant differences between the groups for any demographic variables. At baseline, the YOMI group reported significantly higher levels of anxiety (*p* = .036) and ADM (*p* = .017) than did the control group; it also had significantly higher ADM levels than the Yin yoga group *(p* = .007).

**Fig 1:**
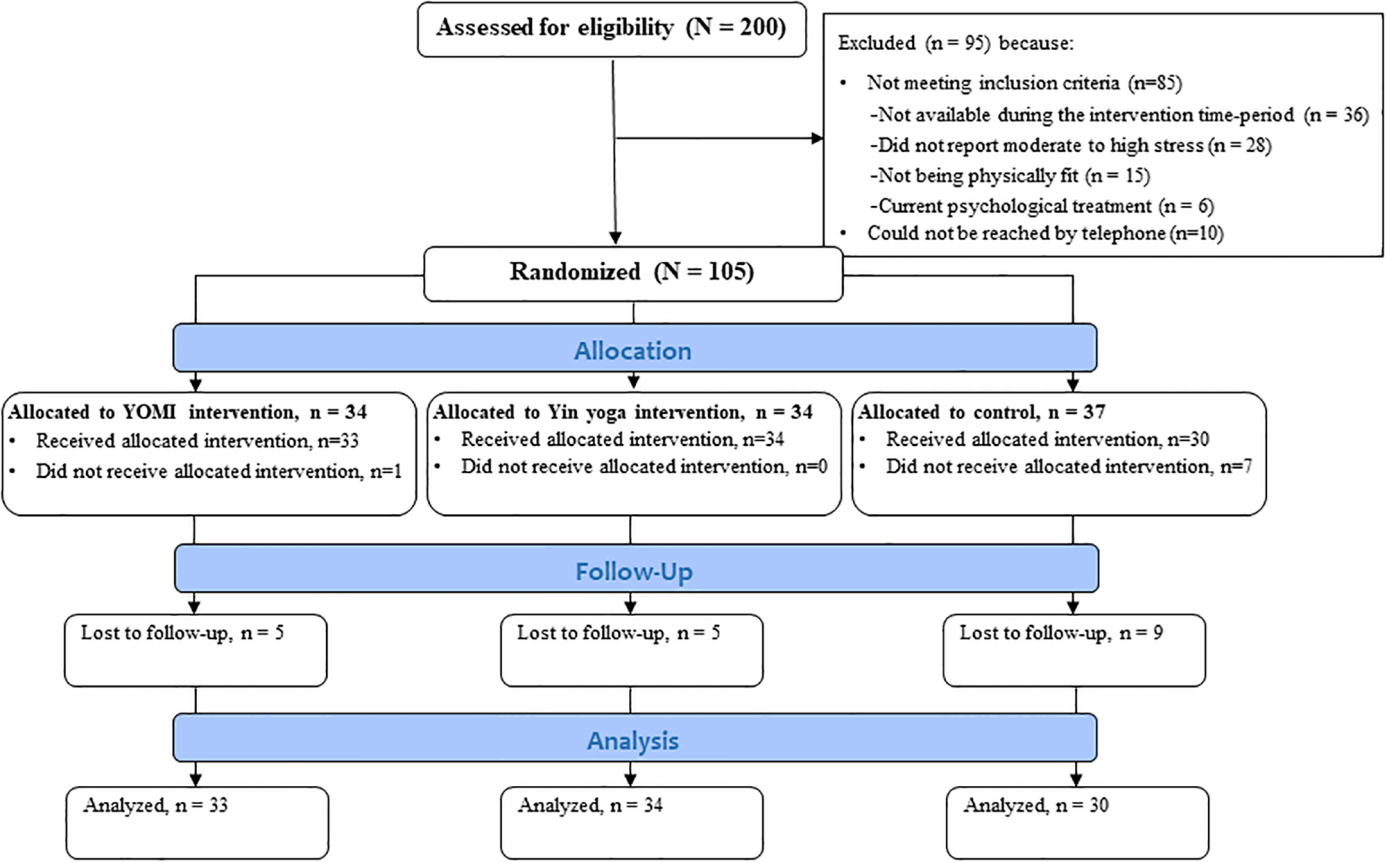
CONSORT flow diagram.

Fig 1 presents the CONSORT flowchart of the different stages of the study. We performed attrition analyses, which revealed that for the YOMI and control groups, those who completed the study did not score significantly differently on any variables at baseline as compared to those who did not complete the study. However, for the Yin yoga group, non-completers reported a significantly higher level of perceived stress than did those who completed both measurements, *t*(32) = -2.19, *p* = .036.

Table 2. shows the means and standard deviations of the study outcomes at the two assessment points.

**Table 2:** Means and standard deviations (in parentheses) on study outcomes at two time points.

Variable	YOMI Group		Yin Yoga Group		Control Group	
	Pre	Post	Pre	Post	Pre	Post
ADM	6.11 (0.30)	5.96 (0.37)	5.83 (0.34)	5.70 (0.31)	5.86 (0.38)	6.16 (0.44)
Perceived stress	20.64 (5.87)	12.75 (4.93)	19.32 (5.69)	14.10 (8.28)	19.17 (6.21)	14.71 (6.60)
Anxiety	11.27 (3.61)	7.11 (3.70)	10.29 (3.66)	6.90 (4.34)	8.87 (4.13)	7.76 (4.02)
Depression	6.36 (3.33)	3.61 (2.85)	6.18 (2.90)	4.41 (3.63)	5.60 (3.27)	3.81 (3.09)
Insomnia	20.24 (5.90)	15.25 (6.36)	20.68 (6.21)	17.07 (7.02)	18.10 (6.68)	16.90 (5.69)

*Note*. ADM = adrenomedullin.

Table 3 shows the correlations between the study outcomes at baseline for the whole sample. The correlations between ADM and the psychological outcomes were generally low and nonsignificant, while the intercorrelations between the psychological outcomes were moderately strong.

**Table 3:** Correlations between the study outcomes at baseline.

	1	2	3	4	5
1. ADM	---	-.038	.066	.150	.055
2. Perceived stress		---	.697***	.552***	.470***
3. Anxiety			---	.569***	.441***
4. Depression				---	.517***
5. Insomnia					---

*Note*. ADM = adrenomedullin.

*** *p* < .001

Finally, we asked participants to report how many minutes they performed their daily breathing exercises. Twenty-three (79.3%) of the 29 completers in the Yin yoga group and 27 (96.4%) of the 28 completers in the YOMI group returned the daily breathing assignment sheets. No significant results were found in total assignment time between the YOMI (*M* = 321.96, *SD* = 198. 64) and Yin yoga (*M* = 238.96, *SD* = 131.19) groups, *t*(48) = 1.71, *p* = .094.

### Pre-post changes in adrenomedullin

We observed significantly greater pre-post reductions in plasma ADM levels (expressed as standardised values) in both the YOMI (*β* = -0.41, *p* < .001) and Yin yoga (*β* = -0.46, *p* < .001) groups (Table 4) compared to the control group. More specifically, ADM levels in the YOMI and Yin yoga groups decreased after the intervention with standard deviations of .41 and .46, respectively. None of the control variables (i.e. age, sex, BMI, baseline levels of cystatin C) in the regression model were significantly associated with the change in ADM. The overall model’s explained variance was *R*^*2*^ = 0.39.

**Table 4:** Results of multiple regression analyses.

	B	SE B	p	β
**Adrenomedullin *R***^**2**^ **= .39**				
Intervention				
**YOMI**	**-0.34**	**0.08**	***p* < .001**	**-.41**
**Yin yoga**	**-0.39**	**0.08**	***p* < .001**	**-.46**
Age	0.02	0.09	ns	.02
Sex	-0.00	0.01	ns	-.05
Cystatin	-0.34	0.33	ns	-.10
BMI	-0.01	0.01	ns	-.12
Constant	-7.14	6.72		
**Perceived stress *R***^**2**^ **= .08**				
Intervention				
**YOMI**	**-3.99**	**1.59**	***p* = .012**	**-.27**
**Yin yoga**	-0.52	1.58	ns	-.04
Age	0.08	0.12	ns	.07
Sex	-0.64	1.83	ns	-.04
Constant	-7.14	6.72		
**Anxiety *R***^**2**^ **= .33**				
Intervention				
**YOMI**	**-3.55**	**0.71**	***p* < .001**	**-.46**
**Yin yoga**	**-2.20**	**0.71**	***p* = .002**	**-.29**
Age	0.10	0.05	*p* = .051	.18
Sex	0.19	0.82	ns	.02
Constant	-6.48	3.02		
**Depression *R***^**2**^ **= .07**				
Intervention				
**YOMI**	**-1.67**	**0.72**	***p* = .021**	**-.25**
**Yin yoga**	-0.23	0.72	ns	-.04
Age	0.27	0.83	ns	.04
Sex	0.03	0.05	ns	.05
Constant	-2.74	2.96		
**Sleep problems *R***^**2**^ **= .33**				
Intervention				
**YOMI**	**-4.53**	**0.90**	***p* < .001**	**-.47**
**Yin yoga**	**-2.71**	**0.90**	***p* = .003**	**-.28**
Age	0.12	0.07	*p* = .066	.17
Sex	0.89	1.04	ns	.08
Constant	-8.06	3.82		

*Note*. ADM = adrenomedullin. p values for ns vary between .211 and .971. Control group is a reference group. B coefficient refers to the expected increase/decrease in the dependent variable for one unit change in the independent variable. β coefficient refers to the number of standard deviation changes we would expect in the outcome variable for a 1 standard deviation change in the predictor variable.

### Pre-post changes in stress, anxiety, depression, and sleep problems

Participants in the YOMI group (*β* = -0.26, *p* = .012) showed significantly greater pre-post reductions in perceived stress levels compared to the control group (Table 4), whereas no significant differences in changes were found for the Yin yoga group (vs. the controls). The overall model’s explained variance was *R*^*2*^ = 0.08.

Similar results were found for depression scores: A significantly greater pre-post reduction in depression scores was found only for participants in the YOMI group (*β* = -0.25, *p* = 0.012) compared to control; the reduction for participants in the Yin yoga group did not significantly differ from that in the control group. The overall model’s explained variance was *R*^*2*^ = 0.07.

Significantly greater pre-post reductions in anxiety levels were found in both the YOMI group (*β* = -0.46, *p* < .001) and the Yin yoga group (*β* = -0.29, *p* = .002) compared to controls. Regarding the control variables, anxiety increased somewhat with age (*β* = 0.18, *p* = 0.051). The overall model’s explained variance was *R*^*2*^ = .33.

We observed significantly greater pre-post reductions in the ISI in both intervention groups compared to controls, with a larger reduction in the YOMI group (*β* = -0.47, *p* < .001) than in the Yin yoga group (*β* = -0.29, *p* = .003). None of the control variables in the multiple regression model were significantly associated with insomnia. The overall model’s explained variance was *R*^*2*^ = .33.

### Relationship between pre-post changes in ADM and psychological variables

We evaluated the correlations between the pre-post reduction in ADM and the pre-post reductions in perceived stress, anxiety, depression, and sleep problems for the entire study population. We found significant but weak correlations (*p* < .05) between pre-post ADM reduction and reduction in depression symptoms and anxiety, but no significant correlations with reductions in sleep problems and perceived stress (Table 5).

**Table 5:** Pearson correlations between pre-post changes on adrenomedullin and psychological measures.

Change in	Adrenomedullin	
	r	p
**Perceived stress**	.16	.19
**Anxiety**	.28	.02
**Depression**	.25	.04
**Sleep problems**	.13	.31

## Discussion

The aim of this randomized controlled trial was to examine whether two 5-week yoga interventions—Yin yoga, and Yin yoga in combination with psychoeducation (the YOMI program)–reduce levels of plasma ADM and improve stress-related psychological health factors in moderate to highly stressed (but otherwise healthy) adults. We also examined whether pre-post changes in ADM were associated with pre-post changes in psychological health factors.

As expected, we found significantly greater reductions in plasma ADM levels, anxiety, and sleep problems in both intervention groups compared to the control group. We also observed significant reductions in depression and perceived stress in the YOMI group. The improvements in mental health correspond with previous research on yoga and health, showing that even relatively short yoga interventions have important health benefits (e.g. [46]). We also expanded on previous findings by showing that Yin yoga practice twice a week for five weeks decreases the biomarker ADM. This result is interesting because high levels of ADM are strongly associated with the development of serious NCDs and premature mortality in the general population, as well as with short-term mortality in acutely ill patients [10, 11]. Previous research has shown that ADM increases under high stress [47, 48] and our finding expands this research by showing that yoga-based interventions can reduce ADM levels.

Contrary to expectations, the pre-post changes in ADM were not significantly related to the pre-post changes in perceived stress levels. This could be explained as an effect of the well-known limitations of self-report measures, as described below. However, it could also mean that ADM is not necessarily directly related to self-rated stress, but rather to clearer manifestations of psychopathology such as anxiety and depression. In support of this, we found significant relationships between changes in ADM and changes in depression, which replicates the findings of previous research [17, 18]. Congruently, there was a significant relationship between changes in ADM and changes in anxiety, in line with the result of one previous study [19]. Conversely, another study found reduced levels of ADM in patients with anxiety disorder [20]. The reason for this difference between studies is unclear, but our study participants reported higher levels of anxiety, had lower BMI, were younger, and were predominantly women, all of which might account for the difference.

Further, in line with previous research [25], our results showed that combining Yin yoga with psychoeducation (i.e. the YOMI program) had better overall health benefits than did practicing Yin yoga alone. This also corresponds with the notion of the interventions working as tools for coping with stress. Apparently, both interventions helped highly stressed participants sleep better and reduced their anxiety, which perhaps suggests that Yin yoga reduces physiological arousal. The health benefits of yoga have previously been suggested to depend on activation of the parasympathetic nervous system [49–52]. The psychoeducation, on the other hand, was meant to provide participants with additional insights into the functions of stress and how it can be coped with through mindfulness (including attention to breathing and the practice of acceptance). The results show that this helped participants to also *feel* less stressed, as well as helped lower their levels of depression.

### Limitations

One major limitation of this study was attrition, which possibly attenuated statistical power and increased the risk of type II error—this was especially likely in the Yin yoga group, as those who discontinued the study reported significantly higher levels of stress at baseline. However, the moderate effect sizes observed for many study outcomes suggest that the changes are noteworthy. Furthermore, participants in this study were recruited using convenience sampling. All individuals were well-educated and screened for pathology, and the majority of participants were women. Therefore, the generalizability of findings is limited, warranting further investigation.

All psychological measures used the self-report method. Although this is common in psychological research, the self-report method suffers from two major limitations: the fact that it relies on conscious access to psychological states, and social desirability concerns. Our study design also lacked a placebo control group, which future studies might wish to include, in order to separate intervention effects from expectations of improvement.

At baseline, the YOMI group reported significantly higher ADM and anxiety than the controls, providing some indication that pre-post differences between the groups might have been influenced by the statistical phenomenon known as “regression to the mean.” However, neither variable was in the extreme range—ADM levels were similar to those for healthy individuals [9] and anxiety levels were moderate [53]. Furthermore, significantly greater pre-post intervention differences in ADM and anxiety were found in both intervention groups, which minimizes the likelihood of the conclusion (i.e. the intervention led to decreased anxiety and ADM) being inaccurate.

Finally, although ADM has been linked to stress, anxiety, depression, NCDs, and increased mortality, it is unknown whether ADM is *causally* linked to these factors. ADM is most likely a marker of various forms of biological stress or illness. Future research is needed to investigate the possible mechanisms explaining the relationship between ADM and mental health.

### Implications for clinicians and policymakers

Since elevated ADM is strongly associated with the development of severe disease and increased mortality [10, 11], the finding that short Yin yoga interventions reduced ADM could have implications for clinicians and policymakers aiming to suggest preventative interventions for improving public health. Although increases in stress-related illness [54, 55] are probably best combatted via societal change—particularly, by attempting to reduce stressors people face—many individuals could also benefit from finding ways to improve coping when faced with unavoidable stressors. This study suggests that incorporating Yin yoga could be an easy and low-cost method of limiting the negative health effects associated with high stress. Complementing Yin yoga with psychoeducation appears to have even stronger effects on psychological health.

### Conclusions

The results of this randomized controlled trial show that a short, five-week Yin yoga-based intervention has positive health effects, particularly in terms of decreasing levels of ADM, a biomarker associated with NCD and premature mortality, and reducing the subjective experience of sleep problems and anxiety in highly stressed adults. Adding psychoeducation to the Yin yoga practice can also lower depression and perceived stress. Preventive actions leading to a reduction in ADM are potentially associated with reduced vascular and cardiac stress as well as a reduced risk of major NCDs.

## Supporting information

S1 File CONSORT checklist. (DOC)

S2 File Project protocol. (PDF)

S3 File Data file. (SAV)

S1 Table Topic of each session of the YOMI program. (PDF)
