# Supplementary material for: Five-week yin yoga-based interventions decreased plasma adrenomedullin and increased psychological health in stressed adults: A randomized controlled trial
Source: PLoS One. 2018 Jul 18;13(7):e0200518. doi: 10.1371/journal.pone.0200518 (PMC6051627; doi:10.1371/journal.pone.0200518)
Supplement: S2 File — (PDF) [file pone.0200518.s002.pdf]

Project plan was approved by the Regional Ethical Review board at Lund University (Dnr 2015/610)

PROJECT PLAN

YOGA PRESCRIPTIONS (YOGA PÅ RECEPT) – STUDY 2

THE EFFECTS OF YOGA ON PSYCHOLOGICAL AND PHYSIOLOGICAL HEALTH IN A POPULATION BASED COHORT

The general purpose of this study is to evaluate the benefits of yoga as a 'mind-body' practice, by measuring its effects on health-related psychological and physiological variables.

The research group, in addition to myself - Daiva Daukantaite - contains the following researchers from the Department of Psychology at Lund University: Una Tellhed, PhD, and Rachel Maddux, PhD, and the following researchers from the Department of Clinical Sciences at Lund University: Thomas Svensson, MD, PhD student, and Olle Melander, MD, Professor.

We also aim to investigate whether a relation exists between subjective (psychological) and objective (physiological) measures, with a particular focus on stress as the primary outcome. Further, we intend to include measures of mindfulness, breathing and experiential avoidance – variables that may explain the mechanism by which yoga exerts its putative health-promoting effect. Similar to our previous research on the topic of yoga and health (Dnr 2014/906 & 2015/247) we expect that changes in stress, anxiety, sleep problems via yoga practice will lead to improved health outcomes among individuals reporting moderate to high levels of perceived life stress.

Background

Stress and worry in everyday life are common features of Western society, wherein demands to constantly achieve and not get ill are high - in the workplace, at school or at home. Evolutionarily, stress is a biological mechanism, necessary for all living creatures when being exposed to threats in the immediate environment, tapping the fight-or-flight-response via activation of the sympathetic nervous system (Kroese, 2000; Wallace, 2009). However, when exposed to stress during prolonged periods of time, the immune system is affected, leading to physiological as well as psychosocial problems (Kroese, 2000). The consequences of untreated high stress can not only turn into reduced job capacity, work roles conflicts, etc., but also into a mental illness such as an anxiety disorder or/and depression, which, in many cases, results in long periods lost from work (Hejbel, 2012).

Stress is caused by many different factors. When a person perceives that the demands upon him/herself are higher than the resources to meet those demands, he/she experiences psychosocial stress (Morone et al., 2012). A way of explaining the psychological aspects of stress is that it emerges in the collision between the individual's expectations and the actual experience of the present moment, creating feelings of being stressed when these do not match (Kroese, 2000). Kroese (2000) also argues that underlying the stress response is usually some sort of worry, often related to feeling a lack of control. Cohen et al. (1983) regards the stress response as not merely dependent on the intensity or number of stressful events, but also on personal and contextual factors. How an individual appraises events occurring in his or her life will affect the level of perceived stress (Cohen et al, 1983; Kroese, 2000).

Different coping strategies may be helpful in managing stress. One of these strategies, acceptance, has been shown to be powerful in helping to ameliorate the stress reaction (Kroese, 2000; Schenström, 2007). Being able to accept that what is going on in the surrounding environment is not always in accordance with one's expectations can be a powerful tool in coping with stress. Acceptance is sometimes interpreted as passive resignation (Baer et al., 2006); however, it has more to do with allowing oneself to see and endure reality as it is, without avoidance or judgments - to accept the full experience while refraining from self-criticism and impulsive reactions (Baer et al., 2006; Kåver, 2004; Gunaratana, 2011). Furthermore, mindfulness, a broader concept that encompasses acceptance, can help change a person's approach toward being able to focus his/her energy on the things that are changeable and within one's control (Kroese, 2000; Schenström, 2007).

One common and recommended strategy to manage stress is to exercise regularly (Camiletti-Moirón et al., 2013). The up-to-date scientific knowledge on how to prevent and treat various diseases and conditions using physical activity is summarized in *Physical Activity in the Prevention and Treatment of Disease* (2010). However, in order to cultivate acceptance, improvements in mindfulness - an important segment in all yoga classes- are needed. In the above mentioned book, yoga is named among the various types of physical activity and exercise; however, the main focus in the majority of yoga classes is on both body and mind, with focused breathing acting as a physiological compass that reflects one's current emotional and mental state (Brown & Gerbarg, 2009). Thus, yoga practice not only elicits physical motion, but it is also a method for cultivating mindfulness and acceptance. Morone et al. (2012) carried out a phenomenological study to identify which themes appeared to be essential in the process of applying mindfulness as a method to cope with psychosocial stressors. The four identified themes were *awareness*, *coping*, *serenity* and *change in perspective*, pointing toward an increased ability in the participants to be self-aware and self-reflective, and being able to stop and focus on the moment instead of ruminating on the past or worrying about the future.

Ross and Thomas (2010) reviewed studies comparing the effects of yoga (i.e., physical activity as a type of mindfulness practice) and exercise (i.e., physical activity without emphasis on mindfulness) on a variety of health conditions. The authors concluded that yoga may be as effective as or better than ordinary exercise at improving a variety of health-related outcomes. Given that yoga is multi-dimensional; that is, it includes aspects of exercise, breath work, concentration, and meditation, it is not surprising that researchers have found positive results in so many different areas (e.g. relieving physical symptoms, lowering blood pressure, improving perceptions of mood and anxiety among others. For review, please see Ross & Thomas, 2010).

To illustrate, exercise has been recognized as a key component in cardiac rehabilitation. Yoga, when added to usual cardiac care in randomized trials, has been shown to be significantly better than usual care at improving blood lipid levels (Manchanda et al., 2000), decreasing markers of inflammation (Pullen et al., 2008), and in reducing the number of revascular procedures (Manchanda et al., 2000). These finding are particularly salient to the present study as the epidemiology of carotid artery disease is the overarching focus of the Malmö Diet and Cancer Cardiovascular Cohort and it's offshoot, the Malmö Offspring Study, from which our participants will be primarily recruited.

Yoga has also shown a positive effect on psychological problems, including depression and anxiety (Kinser et al., 2013). Several studies have shown a significant decrease in stress

levels even over short time periods of yoga practice and mindfulness (Brisbon & Lowery, 2009; Carmody et al., 2009; Chambers Christopher et al., 2006; Cowen & Adams, 2005; Morone et al., 2012; Salmon et al., 2009). Streeter et al. (2010) compared a group of yoga practitioners to a group of people engaging in walking as exercise, showing greater improvement of mood along with greater decreases in anxiety in the yoga group as compared to the walking group. They also found that the behavioral intervention (i.e. yoga) showed a positive association between changes in thalamic GABA levels and improvements in mood and anxiety, compared to the walking group. In sum, the practice of yoga and mindfulness seems to provide individuals with powerful tools to cope with daily stress and related problems.

Aims

The primary purpose of this study is to evaluate the effects of yoga on: 1) psychological health, specifically measured as stress level, stress coping strategies, depression, anxiety, well-being, life harmony, diet, and sleep quality; and 2) physiological health, specifically measured as blood plasma and serum stress markers (e.g., hormones, inflammation), blood plasma stress-related indicators (i.e., metabolic and cardiovascular function), and non-invasive clinical measures of stress-related indicators (i.e, cardiovascular and autonomic nervous system function) including blood pressure, arterial stiffness (via pulse wave velocity), and heart rate variability (via electrocardiogram), as well as digestive health via gut microbiota fecal sampling. Further, we intend to explore whether any changes in coping strategies (breathing, mindfulness) as a result of yoga practice may correspond to predicable changes in well-being.

Method

Participants

We plan to conduct a randomized controlled field experiment. In order to properly evaluate the effects of the intervention statistically, we will recruit approximately 120 individuals (please see power analysis below). Study participants will be recruited primarily from the ongoing Malmö Offspring Study (MOS; Dnr 2012/594) which takes place at the Clinical Research Unit at Skånes Univeristy Hospital in Malmö (Klinisk Forskningsavdelning på Skånes universitetssjukhus i Malmö). MOS is an inter-generational study where all children and grandchildren of individuals in the Malmö Diet and Cancer Cardiovascular Cohort (MDC-CC; n=6103) are invited to participate. The MDC-CC is a population-based study that investigates the epidemiology of carotid artery disease (LU 51-90 and Dnr 2009/633; Melander, et al., 2009, 2012). Individuals participating in MOS who have indicated an experience of chronic stress during the past year will be contacted by the research team. Other individuals visiting the Clinical Research Unit and/or members of the community are also eligible to participate in the study according to the inclusion/exclusion criteria (please see below).

Procedure

<u>1. Telephone and In-Person Recruitment and Screening</u>

Recruitment will be accomplished in two ways, 1) via telephone, and/or 2) in person. Telephone recruitment will be directed to participants in the Malmö Offspring Study (MOS) as described above. For individuals in MOS indicating a chronic experience of stress within the past year, information letters will be sent (see Appendix 3). Then they will be contacted by a person employed by the research project. During the telephone call, information about the current yoga study, as described in the information letter in the Appendix 3 and Appendix 4:1 will be given, and the person will be asked if he/she is interested in participating. The telephone interviewer will also ask questions related to the inclusion/exclusion criteria described below. In-person recruitment will be directed to individuals visiting the Clinical Research Unit at Skånes University Hospital in Malmö who indicate high stress to the personnel. They will be orally given the same information as during the telephone interview previously described. Recruitment will take place over an approximate 2-month period.

Inclusion criteria:

1. A score on four items of the Perceived Stress Scale (PSS; Cohen et al., 1983) between 8-16, thereby indicating a current stress experience of at least moderate level (please see Appendix 5:12).
2. Ability to participate in the intervention during the time-period.
3. Comprehensive in Swedish (the intervention will be in Swedish).

Exclusion criteria

1. Ongoing yoga practice or any regular practice of yoga or meditation within the last 6 months.
2. Any known physical limitations that may prevent light physical activity (yoga postures).

When a person qualifies for the study, an appointment will be scheduled for him/her to come to the Clinical Research Unit at Skånes Univeristy Hospital in Malmö for the initial tests to be taken (see Measures below). The name of each participant will only be registered on a single master list in combination with a numeric code to ensure participant anonymity (please see Ethical Considerations below for further detail). Numeric codes will be used throughout the remainder of the study to identify participants. Additionally, the master list will provide the randomly generated number indicating which study group he/she is assigned to. Each person will be randomized at the individual level to one of the following conditions:

1) Group 1: Stress education + Yoga (SED_YOGA). Education about stress plus yoga practice, 2 times per week for 5 weeks.
2) Group 2: Yoga (YOGA). Yoga practice, 2 times per week for 5 weeks.
3) Group 3: Control (CONTROL). Education about stress plus yoga practice, 1 session after the 5-week main study is completed.

2. First Appointment at the Clinical Research Unit (pre-testing)

At the first appointment, participants will be given a detailed information letter to read about the study and his/her participation (please see Appendix 4:1). Participants will also have the opportunity to ask any questions about the study and his/her participation. He/she will then be given the informed consent document to read and sign (please see Appendix 4:2). When

informed consent is given, a copy will be provided to the participant, and the study measurements will begin.

Measures

Physiological measures:

Physiological measures will be carried out at the beginning of the appointment in order to capture, in particular, fasting blood samples. The full battery of physiological measures entails:

1) Fasting blood sampling (serum and plasma). Blood sampling will be performed via venipuncture which involves extracting venous blood through a small sterile needle and collection in a vacutainer (sterile glass or plastic tube). A total of 3 tubes will be collected for each participant, amounting to a total of 65ml of blood per person. Blood samples will then be sent to a biobank. A biobank is a freezer (-80 degrees) where biological samples are stored (please see Ethical Considerations for further details). Blood analysis will include stress hormones, inflammatory biomarkers, metabolic profiling, cardiometabolic biomarkers, cholesterol, triglycerides, creatinine, cathecolamines (noradrenalin and adrenalin), renin, cortisol, and glucose concentration.

2) Fasting and 120-minute glucose tolerance test. The glucose tolerance test determines how the body responds to glucose (sugar). After a fasting blood sample is taken (see above procedure), participants will be asked to drink a liquid containing glucose. A second blood sample will be taken 120 minutes later.

3) Heart rate variability with electrocardiogram during deep breathing. Heart rate variability is the variation in the time interval between heartbeats. It can be measured with an electrocardiogram (ECG). An ECG is a simple, painless test that involves attaching 12 to 15 soft electrodes to the chest, arms, and legs. These electrodes are attached to electrical leads (wires), which are then attached to the ECG machine. The ECG machine shows the heart's electrical activity as line tracings on paper. Using ECG, blood pressure or arterial pulse recordings can show the interplay between sympathetic and parasympathetic nervous systems.

4) Gut microbiota profiling of fecal samples. Microbes perform essential functions such as digesting food and synthesizing vitamins, and may help in understanding health problems and diseases. Gut microbiota can be measured using a single fecal sample. Participants will be given a container with lid to collect a sample at the hospital during the visit, or alternatively to collect at home and return the following day along with the sphygmomanometer. The fecal sample will also be sent to the biobank.

Psychological measures (Please see Appendix 5:1-5:11 to review scales in full):

1) <u>The Perceived Stress Scale</u> (PSS; Cohen et al., 1983) is a 10-item scale measuring the degree to which situations in one’s life are appraised as stressful. The items (e.g. In the last month, how often have you felt that you were unable to control the important

things in your life?) are rated on a 5-point scale from 0 (never) to 4 (very often). It has shown good internal consistency with alpha coefficients range from .80 to .86 (Nordin & Nordin, 2013).

2) The Hospital Anxiety and Depression Scale (HADS; Zigmond & Snaith, 1983) is used to assess levels of anxiety and depression. It is a fourteen item scale: seven of the items relate to anxiety and seven relate to depression. Items can be scored together for total scale score, or by subscale. Each item is scored from 0-3. The HADS has been used extensively, and it's psychometric properties are well-established (Bjelland, Dahl, Haug, & Neckelmann, 2002). Average alpha coefficients for the HADS-anxiety and depression subscales are .83 and .82, respectively.Fasting concentration of LDL and HDL cholesterol, triglycerides, creatinine, cathecolamines (noradrenalin and adrenalin), renin and cortisol.

3) The COPE Inventory (COPE; Carver et al., 1989) assesses 14 conceptually distinct coping strategies. It includes 13 scales with four items each and one single item question to assess different strategies for coping with stress. All items are reported on a 4-point scale (1 = I usually don't do this at all; 2 = I usually do this a little bit; 3 = I usually do this a medium amount; 4 = I usually do this a lot). The COPE inventory has been tested previously and found to have good psychometric properties (Carver et al., 1989; Stone, Greenberg, Kennedy-Moore & New- man, 1991). We will also incorporate 4 exploratory, yoga-related items to the COPE Inventory: (1) I take several deep, calming breaths, (2) I breathe deeper and calmer, (3), I focus on my breathing, (4) I concentrate on my breath.

4) The Insomnia Severity Index (ISI; Morin, 1993) is designed to be both a brief screening measure of insomnia and an outcome measure for use in intervention research. The index contains seven items including the severity of sleep onset and maintenance (e.g., middle and early morning awakening) difficulties, satisfaction with current sleep pattern, interference with daily functioning, appearance of impairment attributed to the sleep problem, and the degree of concern caused by insomnia. The items are rated on a five-point Likert scale from 0 (not at all) to 4 (extremely). It has shown good internal consistency (Cronbach's alpha of .76; Bastien, Vallie'res, & Morin, 2000). We will also include one exploratory question: With focus on the past week, how many hours did you sleep at night: a) During a typical weekday (Monday to Friday)? b) During Saturday or Sunday?

5) Diet is a 2-item measure that is used as an exploratory questionnaire. The questions are as follows: Have you changed your eating habits since you started your participation in the study? Categorical answer choices include 1) No, not at all, 2) Yes, to some extent, 3) Yes, to a great extent. A second question is open-ended: If you have changed your eating habits, please describe how. This very brief questionnaire will be given only once at the end of the study to explore whether dietary changes have occurred following the 5-week study period.

6) General Health Questionnaire (GHQ; Goldberg & Williams, 1988) consists of 12 items, each one assessing the severity of a mental problem over the past few weeks using a 4-point Likert-type scale (from 0 to 3). These scores generate a total score ranging from 0 to 36. The positive items are corrected from 0 (always) to 3 (never) and the negative ones from 3 (always) to 0 (never). Higher scores indicate worse

health. The instrument is considered as reliable and has been translated into 38 different languages. The reported Cronbach alpha coefficient for the GHQ is a range of 0.82 to 0.86 (Golderberg, & Williams, 1988).

7) The Harmony in Life Scale (HILS; Kjell et al., in press) assesses a global sense of harmony in one's life and consists of 5 statements (e.g., "Most aspects of my life are in balance") for which participants are asked to indicate degree of agreement on a 7-point Likert scale (1 = strongly disagree, 7 = strongly agree). The harmony score is established by summarizing the 5 statements for each participant. It has shown high internal consistency (Cronbach's alpha of .91; Garcia et al., 2013).

8) The Life Satisfaction Scale (LSS) consists of three items rated on a 7-point scale. The scale has previously been reported to have good internal consistency (α = .90; Ahlerup, 2015).

9) The Kentucky Inventory of Mindfulness Skills -Short Version (KIMS; Baer, Smith, & Allen, 2004; Höfling, Ströhle, Michalak, & Heidenreich, 2011) is a 20-item multi-dimensional scale of interrelated skills related to what one does while practicing mindfulness, and how one does it. The "what" skills include observing (noticing or attending to) current experience, describing (noting or labeling observed experiences) with words, and participating (focusing full attention on current activity); the "how" skills include being nonjudgmental (accepting, refraining from evaluation), being one-mindful (using undivided attention), and being effective (using skillful means; Baer et al., 2008). The subscales show good internal consistency with alpha coefficients ranging from .80 to .85.

10) The Brief Experiential Avoidance Questionnaire (BEAQ**;** Gámez et al., 2014) consists of 15 items used to assess behavioral avoidance (situational avoidance of physical discomfort and distress), distress aversion (non-acceptance of or negative attitudes toward distress), procrastination (delaying activities that may cause distress), distraction/suppression (attempts to ignore or suppress distress), repression/denial (distancing and dissociating from distress), and distress endurance (willingness to engage in behavior that is consistent with one's values even when in distress). Items are rated on a 6-point scale ranging from 1 (*strongly disagree*) to 6 (*strongly agree*). The scale shows evidence of good internal consistency with alpha coefficients ranging from .80 to .89 (Gámez et al., 2014).

11) The Yogic Breathing as Stress Coping Scale. We constructed a 4-item measure, the Yogic Breathing as Stress Coping Scale (YBSC), to evaluate how often individuals use breathing as a coping strategy when they experience a stressful event. The instruction, *What do you generally do when you experience stressful events?* was adapted from the COPE scale (Carver et al., 1989). The four items are as follows*: I take several deep, calming breaths; I breathe deeper and calmer; I focus on my breathing; I concentrate on my breath.* The four items are rated on a five-point Likert scale from 1 (I usually don't do this at all) to 5 (I usually do this a lot).

Participants will be asked to wait (if needed) until a total of 2 hours have passed since their fasting glucose tolerance test, so that a 120-minute measurement of plasma glucose concentration can be acquired. Following this, participants will be invited to a quiet area/room where breakfast will be provided free of charge. After breakfast, participants will

complete the psychological measures and be instructed by a Clinical Research Unit staff member on how to use the sphygmomanometer described above (the portable, digital blood pressure machine). Participants will also be given a container with lid to collect a fecal sample at home. Prior to leaving their appointment, participants will be given an information letter about their yoga practice (Please see Appendix 4:3-4:5), and they will be given a free yoga mat to bring to class (exception: Control group participants will receive their yoga mat during the workshop, please see below). Participants are asked to return the sphygmomanometer and fecal sample to the Clinical Research Unit the following day.

3. Yoga Interventions – 5 weeks

Group 1: Stress education + Yoga (SED_YOGA)

Stress education + Yoga brings together education about stress, mindfulness and yoga practice. It will involve weekly group meetings, homework (please see Appendix 4:6), and yoga postures. Stress education and mindfulness will make up one portion of the intervention. This will take place in a group lecture format and shall be conducted by a mental health professional(s). Yoga practice in a group format will make up the second portion of the intervention. The word yoga means 'to yoke' or 'unite' in the sense that it is a practice of connecting the mind with the body. Yoga thus focuses on mental and physical strength building exercises and postures, and in this study, will be lead by a trained yoga instructor(s). More specifically, yoga practice will involve controlled breathing, prescribed body positions, and meditation, with the goal of attaining a state of relaxation. Instructors will lead yoga practitioners in and out of yoga postures (poses) verbally throughout the sessions. Stress education + Yoga will be held 2x/week in the evenings for 5 weeks.

Group 2: Yoga only (YOGA)
The Yoga only group will include the same yoga practice as described above for Group 1, but it will not include the stress education component.

Group 3: Control (CONTROL)
The control group will not participate in any yoga practice for the duration of the research study. However, because these participants had indicated moderate to high levels of stress during study recruitment, we want to provide them with an introduction to yoga, after the study is completed. Therefore, each control group participant will be invited to a 3-hour yoga workshop directly following the 5-week study period. This workshop will include education about stress and mindfulness as well as an introduction to yoga practice. Similarly to Groups 1 and 2, participants in the Control group will receive a free yoga mat.

Approximately one week before the yoga intervention is due to end, participants will receive a letter by mail indicating their second appointment time at the Clinical Research Unit (please see Appendix 4:7-4:8).

4. Second Appointment at Clinical Research Unit (post-testing)

At the follow up measurements, after the 5-week yoga practice, the participants will again be invited to the Clinical Research Unit at Skånes University Hospital in Malmö and have the same physiological measures taken as in the the first appointment. They will also fill out the same survey with psychological measures as they did at the first appointment. Breakfast will

again be offered following completion of the measures. Finally, participants will be thanked for their participation in the research study.

Required sample size, a priori power analyses and statistical analyses

The main statistical analyses will be regression analyses by estimating the models with full-information maximum likelihood (FIML) in AMOS software allowing to perform Intent-To-Treat analyses. The outcome variables will be all study variables calculated as the difference between pre-test and post-test scores.

Mediation models will be tested with the macro PROCESS by Preacher and Hayes (2008) which implements a bootstrapping procedure to create confidence intervals for indirect effects in the mediation model.

G*Power, 3. 1. 7 (Faul, Erdfelder, Lang, & Buchner, 2007) was used to calculate a priori power for analyzing main effects. Assuming that the effect is medium size ($f = 0.25$; see Cohen, 1988), a significance level is of $\alpha = .05$, and the power values of the F tests are .95, a total of $N = 105$ must be recruited.

Ethical Considerations

This research will follow the ethical guidelines regarding informed consent and confidentiality as specified by etikprövningslagen (2003:460). All participants will be informed of the study's aim and method, both orally and in writing, as well as sign an informed consent document prior to participation (see Appendix 4:1-4:2). They will be informed that their participation is completely voluntary and that they can discontinue participation at any time, without giving a reason. Contact information for the project leader will be provided to answer any questions.

The name of each participant will be registered on a single master list in combination with a numerical code. The list will be kept in a locked closet at the Clinical Research Unit at Skånes University hospital in Malmö, stored apart from other study documents. Numeric codes will be used throughout the research on all study documents to identify participants, in order to preserve confidentiality. Once data have been collected, the list with participants' names will be destroyed, ensuring that the identity of the participants will remain undisclosed. No individual names will ever be included in the data file. All study documents will be kept in locked closets, and contain no person names. The data used to assess the inclusion / exclusion criteria in the study (see above), will not be saved for participants excluded from the study. Any data collected from participants choosing to discontinue the study will be destroyed.

Bio-banking and laboratory analysis: Region Skåne biobank BD53 (phone 040-33 10 00) will be the biobank used in this study to store biological samples (i.e., blood serum and plasma, fecal samples). The biobank utilizes a freezer (-80 degrees) where biological samples are kept frozen until the time of analysis. Samples are not marked with a person's name or social security number, but with a numerical code that can only be connected to the participant (described above). Biological samples will be subsequently analyzed at the Department of Clinical Sciences, Faculty of Medicine, Lund University. The person responsible for bio-banking and laboratory analysis of biological samples is Olle Melander, MD, Clinical Research Unit, Department of Clinical Sciences, Skåne University Hospital, Malmö, T: 040-

39 12 09.

One possible risk of participating in this research is that individuals may experience some distress in participating in the study measurements. Blood sample collection and glucose tolerance testing require participants to come to their appointment in a fasting state. This may cause some people to experience hunger and/or fatigue. To minimize discomfort, appointments are made in the early morning and blood collection will be among the first procedures performed. Participants will receive breakfast for free after the study measurements are complete.

Another possible risk is that some participants may experience discomfort with the venipuncture procedure. To minimize this risk, blood collection will only be performed by licensed medical staff at the Clinical Research Unit, Skånes University hospital.

Fasting and 120-minute glucose tolerance test requires participants to drink a liquid containing glucose (sugar) between their first (fasting) and second (120-minute) blood samples. This may cause some participants to feel nervous or anxious about ingesting a liquid. However, the drink is merely sweet, and there are no known risks from participating in a glucose tolerance test.

An electrocardiogram will be performed by a member of the Clinical Research Unit, which may cause some people to feel nervous or embarrassed about having electrodes attached to their upper torso and limbs. However, the electrical signals that are recorded by the machine in reading the activity of the heart are painless. It only requires the participant to lie down and remain still for a few minutes.

Participants may also experience some anxiety or embarrassment about having to produce a fecal sample. However, this is a normal bodily function that causes no harm. Participants will be given the option of giving the sample while at the hospital or, alternatively, in the privacy of their home (and returning the sample the following day). Staff at the Clinical Research Unit are routinely handling biological samples, so it will not appear as an abnormal process.

Participants will be informed about all abovementioned physiological study procedures during the recruitment and informed consent process. They are free to decline participation in this study for any reason. Participation is completely voluntary.

Participants will receive feedback on their results for all physiological measurements that have clinically accepted parameters. This includes information on blood pressure, plasma glucose & lipids and electrocardiogram results. Participants will not receive feedback on exploratory measures since there are no established clinical parameters. This includes all blood sampling procedures, pulse-wave velocity, heart-rate variability and gut microbiota fecal sampling. This information will be made known in the recruitment and informed consent process.

Filling out psychological measures may cause distress for some participants. If this occurs, participants have contact information to the project leader who can answer questions about the study. Further, they will be filling out the questionnaires at the Clinical Research Unit at Skånes University hospital. Should any mental distress occur to the point of requiring emergency assistance, the Unit staff (which includes licensed nurses and physicians) can

refer the person to the Emergency Department at the hospital. The risk for severe distress while filling out questionnaires however is probably low. The measures selected for the study are well-validated and have been used in many previous studies.

Computer Security: Participants will be asked to use a University secured computer to complete psychological measures. Computers are property of the research team. Psychological measures on the computers are configured to a private web-survey that is password protected and only the research team can access. Participants will log-in with their study numeric code and not any other identifying information. When data are transmitted through the web-survey from the participant to the research team, they will be stored on a computer that is not connected to the internet.

Another possible risk in the study is that participants may physically hurt themselves during yoga practice. To limit this risk, participants with any known physical limitations that may prevent light physical activity (yoga postures) will be excluded from participation (please see Exclusion criteria). Also, as the yoga sessions in this study are at the beginners' level, led by a trained yoga teacher(s) and suitable for novice practitioners, we perceive the risk for physical injury to be small.

Last, it is possible that the participants assigned to the Control group (i.e. those who will not receive stress education and/or yoga during the 5-week study period) will be dissatisfied. We will explain to the participants the methodological necessity of having a Control group, to be able to assess the health benefits of yoga methodologically. We will also arrange for a 3-hour workshop for Control group participants, directly after the study period, which will provide them with stress education, mindfulness and yoga practice, as well as a free yoga mat.

References

Baer, R. A., Smith, G. T., Hopkins, J., Krietemeyer, J. & Toney, L. (2006). Using Self-Report Assessment Methods to Explore Facets of Mindfulness. *Assessment,* 13, 27-35.

Bastien, C. H., Vallie'res, A, & Morin, C. M. (2000). Validation of the Insomnia Severity Index as an outcome measure for insomnia research. *Sleep Medicine, 2,* 297–307.

Bellg, A. J., Borrelli, B., Resnick, B., Hecht, J., Minicucci, D. S., Ory, M. et al. (2004). Enhancing treatment fidelity in health behavior change studies: best practices and recommendations from the NIH Behavior Change Consortium. *Health Psychology, 23,* 443-451.

Bjelland, I., Dahl, A. A., Haug, T. T., & Neckelmann, D. (2002). The validity of the Hospital Anxiety and Depression Scale: An updated literature review. *Journal of Psychosomatic Research, 52,* 69-77.

Brown, R. P. & Gerbarg, P. L. (2009). Yoga Breathing, Meditation, and Longevity. *Longevity, Regeneration, and Optimal Health.* 1172, 54–62. doi: 10.1111/j.1749-6632.2009.04394.

Camiletti-Moirón, D., Aparicio, V. A., Aranda, P., & Radak, Z. (2013). Does exercise reduce brain oxidative stress? A systematic review. *Scandinavian Journal of Medicine & Science in Sports, 23,* e202-e212.

Carver, C. S., Scheier, M. F., & Weintraub, J. K. (1989). Assessing coping strategies: A theoretically based approach. *Journal of Personality and Social Psychology, 56(*2), 267-283.

Hejbel, B. Return to work after long-term sick leave: Barriers and facilitators. Doctoral thesis.

Gamez, W. et al. (2014). The Brief Experiential Avoidance Questionnaire: Development and Initial Validation. Psychological Assessment, 26(1), 35-45.

Gámez, W., Chmielewski, M., Kotov, R., Ruggero, C., & Watson, D. (2011). Development of a measure of experiential avoidance: The Multidimensional Experiential Avoidance Questionnaire. Psychological Assessment, 23, 692–713. doi:10.1037/a0023242

Golderberg, D., & Williams, P. (1988). A user's guide to the General Health Questionnaire. Windsor, UK: NFER-Nelson.

Grilley, P. (2002). *Yin Yoga - Outline of a Quiet Practice.* Oregon: White Cloud Press.

Gunaratana, B. (2011). *Mindfulness in plain English*. Somerville: Wisdom Publications.

Kinser, P. A., Bourguignon, C., Whaley, D., Hauenstein, E., & Taylo, A. G. (2013). Feasibility, acceptability, and effects of gentle Hatha yoga for women with major depression: Findings from a randomized controlled mixed-methods study. *Archives of Psychiatric Nursing 27*, 137–147.

Kjell, O.N.E., Daukantaitė D., Hefferon, K., & Sikström, S. (in press). Expanding the conceptualization and measurement of the cognitive component of subjective well-being. *Social Indicators Research.*

Kåver, A. (2004). *Att leva ett liv, inte vinna ett krig - om acceptans*. Stockholm: Natur och Kultur.

Lindgren, J. & Norberg U. (2012). *Teacher Training: Yin Yoga I.* Stockholm: OM Yoga.

Manchanda, S. C., Narang, R., Reddy, K. S., et al. (2000). Retardation of coronary atherosclerosis with yoga lifestyle intervention. Journal of the Association of Physicians of India, 48, 687–694.

Melander, O., et al. (2012). Plasma proneurotensin and incidence of diabetes, cardiovascular disease, breast cancer, and mortality. Journal of the American Medical Association (JAMA), 308(14), 1469-75.

Melander, O. et al., (2009). Novel and conventional biomarkers for prediction of incident cardiovascular events in the community. Journal of the American Medical Association (JAMA), 302(1), 49-57.

Mithoefer, B. (2006). *The Yin Yoga kit - The Practice of Quiet Power.* Vermont: Healing Arts Press.

Morin, C. M. *Insomnia: psychological assessment and management.* New York: Guilford Press; 1993.

Neff, K. D. (2003). Development and validation of a scale to measure self-compassion. *Self and Identity*, 2, 223-250.

Physical Activity in the Prevention and Treatment of Disease (2010). Retrieved from: http://www.fyss.se/wp-content/uploads/2011/02/fyss_2010_english.pdf

Pullen, P. R., Nagamia, S. H., Mehta, P. J., et al. (2008). Effects of yoga on inflammation and exercise capacity in patients with chronic heart failure. *Journal of Cardiac Failure, 14*, 407–413.

Ross, A. & Thomas, S. (2010). The health benefits of yoga and exercise: A review of comparison studies. *The Journal of Alternative and Complementary Medicine, 16*, 3-12. http://www.fyss.se/fyss-in-english/

Stone, A. A., Greenberg, M. A., Kennedy-Moore, E., & Newman, M. G. (1991). Self- report, situation-specific coping questionnaires: What are they measuring? *Journal of Personality and Social Psychology, 61*(4), 648-658.
