# Supplementary material for: Five-week yin yoga-based interventions decreased plasma adrenomedullin and increased psychological health in stressed adults: A randomized controlled trial
Source: PLoS One. 2018 Jul 18;13(7):e0200518. doi: 10.1371/journal.pone.0200518 (PMC6051627; doi:10.1371/journal.pone.0200518)
Supplement: S1 Table — (PDF) [file pone.0200518.s004.pdf]

S1 Table. Topic of each session of the YOMI program

Session		Topic	Summary of content
Week 1	Session 1	Presentation and introduction	**Psychoeducation:** Introductory lecture to yoga and mindfulness, stress, yin and yang.<br>**Mindfulness:** Short story about mindfulness.<br>**Yin yoga:** Practice introducing the yin yoga program and different asanas.
	Session 2	Observation and breathing	**Psychoeducation:** Lecture on the breath, its physiology and function during stress and worry. Introduction to an observational mindset.<br>**Mindfulness:** Breathing exercise 'counting breath', body scan with physical tension and relaxation.<br>**Yin yoga:** Practice focused on breathing and observing thoughts, emotions and bodily sensations.
Week 2	Session 3	Our five senses.	**Psychoeducation**: Introduction to our five senses. Lecture on non-reactive observation and theory of grasping-rejecting.<br>**Mindfulness:** Breathing exercise 'ujjayi pranayama'. Co-breathing exercise. Mindfulness eating exercise focusing on exploring all five senses.<br>**Yin yoga:** Practice focusing on observing all five senses, physical aspects of yin yoga.
	Session 4	Balance	**Psychoeducation:** Lecture on finding balance between activity and restoration, the sympathetic and parasympathetic nervous system. Introducing the three emotion regulation systems and the concept of function and form.<br>**Mindfulness:** Breathing exercise 'Nadi Shodhana'. Exercise 'Three circles'.<br>**Yin yoga**: Practice focusing on self-compassion, using the concepts of function and form.
Week 3	Session 5	YOMI-practice	**Psychoeducation:** recap of lectures from session 1-4<br>**Mindfulness:** Meditation on breathing and letting go.<br>**Yin yoga:** Practice focusing on exploring one's practice.
	Session 6	Introduction to acceptance	**Psychoeducation:** Lecture introducing the definition of acceptance, true/false acceptance, introducing defusion.<br>**Mindfulness:** Mindfulness exercise on observing automatic thoughts. Meditation on passing clouds. Meditation on acceptance.<br>**Yin yoga:** Practice focusing on acting non-reactive and non-judgmental.
Week 4	Session 7	Applications of acceptance	**Psychoeducation:** Lecture on acceptance, applications of acceptance, functional contextualism and compassion. Introduction of "SOAS".<br>**Mindfulness:** SOAS. Compassion meditation

			**Yin yoga:** Practice focusing on acceptance, letting go, using SOAS and compassion.
	Session 8	Self-care	**Psychoeducation:** Lecture on self-care, the concepts of form and function. Introduction of tactile touch as calming tool.<br>**Mindfulness:** Tactile massage, self compassion meditation<br>**Yin yoga:** Practice focusing on exploring self-care using self-compassion and previously introduced tools.
Week 5	Session 9	YOMI practice	**Psychoeducation**: recap of lectures from session 6-8<br>**Mindfulness:** Meditation on conscious breath and happiness<br>**Yin yoga:** Practice focusing on silence and being in stillness
	Session 10	YOMI in the everyday life.	**Psychoeducation:** Lecture on how the YOMI practice can be used in the everyday life. Introducing the concept of ‘monkey mind’.<br>**Mindfulness:** Walking meditation<br>**Yin yoga:** Practice focusing on combining all tools and concepts of the program
